# Using collaborative logic analysis evaluation to test the program theory of an intensive interdisciplinary pain treatment for youth with pain‐related disability

**DOI:** 10.1002/pne2.12018

**Published:** 2020-04-23

**Authors:** Karen Hurtubise, Astrid Brousselle, Chantal Camden

**Affiliations:** ^1^ Faculté de Médecine et Sciences de la Santé Université de Sherbrooke Sherbrooke QC Canada; ^2^ School of Public Administration University of Victoria Victoria BC Canada; ^3^ CanChild Centre for Childhood Disability Research McMaster University Hamilton ON Canada

**Keywords:** interdisciplinary pain rehabilitation program, intervention theory, logic analysis, logic model, pediatric chronic pain, theory‐based evaluation

## Abstract

Intensive interdisciplinary pain treatment (IIPT) involves multiple stakeholders. Mapping the program components to its anticipated outcomes (ie, its theory) can be difficult and requires stakeholder engagement. Evidence is lacking, however, on how best to engage them. Logic analysis, a theory‐based evaluation, that tests the coherence of a program theory using scientific evidence and experiential knowledge may hold some promise. Its use is rare in pediatric pain interventions, and few methodological details are available. This article provides a description of a collaborative logic analysis methodology used to test the theoretical plausibility of an IIPT designed for youth with pain‐related disability. A 3‐step direct logic analysis process was used. A 13‐member expert panel, composed of clinicians, teachers, managers, youth with pain‐related disability, and their parents, were engaged in each step. First, a logic model was constructed through document analysis, expert panel surveys, and focus‐group discussions. Then, a scoping review, focused on pediatric self‐management, building self‐efficacy, and fostering participation, helped create a conceptual framework. An examination of the logic model against the conceptual framework by the expert panel followed, and recommendations were formulated. Overall, the collaborative logic analysis process helped raiseawareness of clinicians’ assumptions about the program causal mechanisms, identified program components most valued by youth and their parents, recognized the program features supported by scientific and experiential knowledge, detected gaps, and highlighted emerging trends. In addition to providing a consumer‐focused program evaluation option, collaborative logic analysis methodology holds promise as a strategy to engage stakeholders and to translate pediatric pain rehabilitation evaluation research knowledge to key stakeholders.

## Graphical abstract

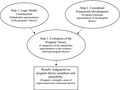

## 1 INTRODUCTION

Pain‐related disability affects eight percent of youth.^1^, ^2^ Within the pediatric pain context, pain‐related disability is defined as pain which impairs youth's ability to perform age‐appropriate activities relevant to daily life.^3^, ^4^ Due to the complexity of these impairments, intensive interdisciplinary pain treatment (IIPT), a specialized multidisciplinary rehabilitation intervention, is viewed as the treatment of choice.^5^, ^6^, ^7^, ^8^, ^9^ To be considered an IIPT program, three or more disciplines (eg, pain specialist, psychologist, physiotherapist) must work together, in an integrated manner, guided by a shared rehabilitation philosophy.^7^, ^10^, ^11^ The aim of IIPT intervention is self‐management, whereby youth and their parents actively engaged in managing pain, and resume participation in age‐appropriate activities.^12^ Although these programs exist worldwide, their comparison and reproducibility are complicated by poor descriptions of the intervention components, and a lack of transparency in how the components produce the anticipated outcomes.^12^, ^13^ Moreover, stakeholders’ perceptions of the value of these programs are missing from the evidence, rendering judgment of their worth difficult.

Integrated knowledge translation (IKT) is a model of collaborative research, where researchers and stakeholders engage together to produce mutually beneficial research and optimize healthcare delivery.^14^ Stakeholder engagement is increasingly recognized as essential and believed to increase accountability, broaden the underlying value base, and enhance the relevance and utilization of the research findings.^15^, ^16^ However, how best to engage stakeholders is less well known. To date, stakeholder engagement in the evaluation of interventions, like IIPT, has been limited.^16^, ^17^, ^19^

Interventions like IIPT are recognized as complex. According to the Medical Research Council, a complex intervention is described as one that contains several interacting components, requires various behaviors to be exhibited by both those delivering and those receiving it, incorporates different groups and organizations, and includes many different outcomes, all the while exhibiting flexibility or tailoring.^18^ The interaction of these multiple components can be represented as a program theory, defined as the specific activities by which an intervention achieves its anticipated outcomes.^20^ Furthermore, it can be illustrated by a logic model, a visual map of this theory.^21^ Stakeholders have unique experience and knowledge of the contextual factors, and how these may have influenced the implementation of an intervention.^22^ Without creating an in‐depth understanding of how complex interventions work and under what condition, treatment outcomes become difficult to explain and are poorly understood.^23^ Currently, an explicit theorization of IIPT and its context is lacking in the pediatric pain‐related disability intervention literature.^12^

Theory‐based evaluation is an approach that may facilitate stakeholder engagement.^24^ It aims to explain how and why programs work (or fail) in different contexts and for different stakeholders.^24^ Logic analysis, a relatively new theory‐based evaluation methodology, theorizes a program by mapping the links between the intervention components and the anticipated outcomes (ie, program theory), highlights contextual influences, and evaluates the plausibility of the program theory against existing evidence and experiential knowledge.^25^, ^26^ Logic analysis uniqueness lies in its theoretical examination of the core intervention characteristics, which must be present to achieve the desired outcomes, and in its identification of the critical conditions necessary for implementation and production of these outcomes.^25^ It is useful in uncovering causal pathways that may be discernible but not always perceptible.^27^ Furthermore, it helps reduce uncertainty about the program theory inherent to complex interventions, provides a preliminary evaluation of the theoretical and empirical foundation of the intervention, and is valuable in recognizing the strengths, weaknesses, and areas of improvement in the program theory.^25^, ^26^, ^28^ Evaluations, using logic analysis, have yet to be applied in pediatric health or rehabilitation interventions, such as IIPT. Furthermore, some methodological gaps exist, including how to engage stakeholder.^29^

In an attempt to broaden the application of this evaluation approach in pediatric health and rehabilitation, this article aims to provide details on the logic analysis methodology including the strategies targeting stakeholder inclusion, the data collected, and the analyses used. To do so, we will present an example of its application in a preliminary evaluation of an implemented IIPT for youth with pain‐related disability and share the findings assessing whether this IIPT was theoretically designed to achieve its desired outcomes.

## 2 METHODS

### 2.1 Study context

With funding from a large philanthropic donation, the IIPT in Western Canada was conceived in response to a growing number of youth presenting with pain‐related disability. This cohort‐based IIPT was influenced by the day‐hospital model described by Logan et al.^9^, ^30^ The 6‐hour daily IIPT operated 5 days per week in a day‐hospital setting and included individual, and group psychology, physical, family, occupational, art, music, and recreation therapies, as well as classroom time with a qualified teacher. Weekly nursing and physician consultations were also incorporated. All providers had specific training and experience working with youth with pain‐related disability. Activities emphasized self‐management knowledge acquisition and skill development, with a focus on restoring function and returning to age‐appropriate activities. Treatment intensity and frequency, the disciplines involved, and the discharge timeframe were individualized and contingent on the achievement of patient‐identified goals established at treatment commencement. Participants received on average 119 hours of scheduled treatment, with an average length of stay of 5 weeks. Once implemented, an evaluation was requested by decision‐makers to determine the program's value and to identify any improvement recommendations.

### 2.2 Study design

To determine whether the core intervention components and critical contextual conditions were present to produce the desired outcomes, a direct logic analysis was used.^26^, ^27^, ^29^ This evaluation was part of a larger participatory study for which ethical approval was obtained.

### 2.3 Participants

An expert panel of representatives from stakeholders involved in the treatment designed for youth with pain‐related disability was identified by facility leadership and recruited via email invitation. The 13‐member panel consisted of five clinicians, a program coordinator, and healthcare manager, all of whom had experience (range 2‐15 years) treating youth with pain and/or disability (eg, pain‐related disability, cerebral palsy). Also included were two teachers with over 10 years of experience academically supporting youth with an array of physical and mental health conditions, two youth managing pain‐related disability, and their parents. As no standards exist to guide the appropriate number of stakeholders to engage in a panel, guidance was gleaned from the consensus building literature, where a diverse group of 5‐15 participants is recommended.^31^, ^32^, ^33^

### 2.4 Procedures

**FIGURE 1:**
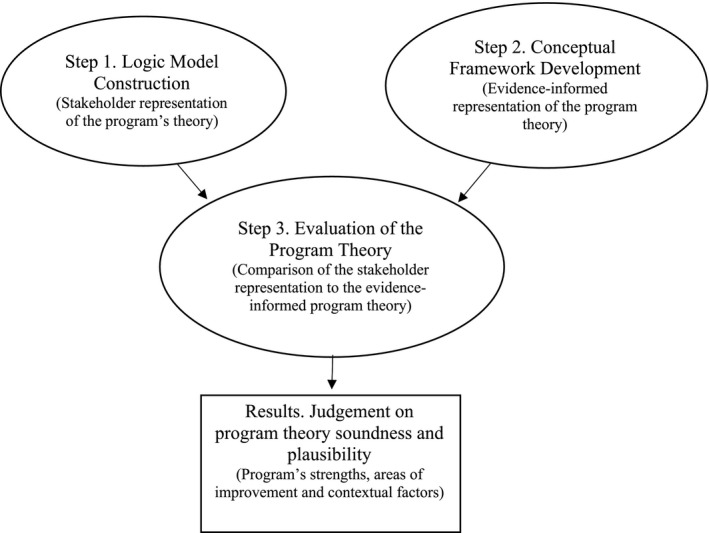
Association between the logic analysis steps and results

To foster an environment conducive to stakeholder engagement, several activities preceded the evaluation process. First, a charter of the role and responsibilities was created and, once agreed upon, was signed by all expert panel and research team members. Additionally, educational resources and training sessions associated with the logic analysis methodology were provided (eg, logic model creation, scoping review processes). The 3‐step logic analysis process described by Brousselle & Champagne^26^ was then followed (see Figure 1).

Table 1 provides a summary of the processes and procedures used in each sequential step. Additional details for each step are provided below.

**TABLE 1:** Summary of logic analysis steps, processes, and procedures

Logic model methodology		
Steps	Process	Procedures
1. Logic model construction: Create a representation of the intervention's program theory and the links between resources, activities processes, and anticipated outcome, using diverse data sources (Brousselle & Champagne, 2011)^26^	Review of all historical program document	Deductive analysis using data extraction form based on logic model components by research team
	Expert panel electronic survey	Deductive analysis using data extraction form based on logic model components by research team
	Draft logic model created by research team using data gathered in documents and surveys	
	Group discussion Validate the primary program objective Review and modify anticipated outcomes (short, medium, and long term) Review and modify resources, activities, and processes Review and modify reach and important contextual factors Establish perceived links between components and anticipated outcomes Achieve agreement on final logic model	Updates of the draft logic model after each meeting by research team. Each subsequent draft returned to expert panel members for further discussion and detailing until agreement achieved.
	Agreement reached by the expert panel members on the logic model representation	
2. Conceptual framework development: Identify and examine the evidence, and document the mechanisms similar to those attributed to the intervention, providing a representative synthesis of the most recent knowledge in the most relevant and meaningful fields of research (Brousselle & Champagne, 2011)^26^	Scoping review framework (Levac et al., 2010)^36^ Identify research question Identifying relevant studies Study selection Charting the data Collating, summarizing, and reporting the results Consultation	Expert panel discussion conducted to identify and achieve agreement on the research question and the study inclusion and exclusion criteria. Studies identified by the research team. Final selection presented to expert panel for approval. Data extracted and deductive analysis completed by research team using a form based on the logic model components and the primary program objective. Draft conceptual framework created by research team and presented to the expert panel for discussion and validation. Expert panel consulted throughout the scoping review process and assisted in the re‐interpretation of the findings in the context of IIPT
	Agreement reached by the expert panel on the interpretation of the conceptual framework	
3. Evaluating the program theory: Review the logic model in light of the evidence contained in the conceptual framework, highlighting the intervention's strengths, weaknesses, and recommendations for improvement (Brousselle & Champagne, 2011)^26^	The logic model was compared to the evidence contained in the conceptual framework for convergence (ie, IIPT strengths) and divergence (ie, IIPT weaknesses and gaps)	A list of strengths, weaknesses, and gaps of the IIPT was identified by the research team, IIPT improvement recommendations formulated, and presented to the expert panel for discussion. Following discussion, only improvement recommendations upon which consensus among the expert panel members was achieved were presented to the hospital leadership team.

#### 2.4.1 Step 1. Logic model construction

In this first step of the 3‐step logic analysis methodology, three data collection methods were used to generate the data required to construct a stakeholder representation of the logic model. These included document analysis, stakeholders’ surveys, and group discussions. All available historical documents (see Table 2 for full list) were analyzed. A stakeholder survey was developed by the research team guided by the semi‐structured interview question for constructing a logic model proposed by Gugiu and Rodriguez‐Campos^34^ (see Appendix S1). Once developed, it was distributed electronically to the expert panel to supplement the document data. A form, founded on the logic model components and their definitions, was used for data extraction of the documents and a deductive analysis followed.^35^ The same process was then repeated for the survey data. The extracted data from the document and the survey analysis were used to populate the various components (ie, resources, research, activities, process, outcomes, contextual factors) of a draft logic model. Six group meetings with the expert panel, facilitated by a member of the research team, were held for the purpose of gathering missing information about logic model components and to clarify inconsistencies. Using various communication strategies (eg, face‐to‐face, FaceTime, telephone, and email), all expert panel members participated in all six discussions. More specifically, at the first meeting, the program goal and objectives were discussed. A dialogue updating each logic model component, the linkages between the components, and the influential contextual features followed in the five subsequent meetings (see Table 1). New iterations of the logic model, based on expert panel feedback, were distributed between meetings, and the iterative process continued until agreement was reached. The sixth iteration was adopted.

**TABLE 2:** Document and survey analysis

Data sources	Document title (y)	Program logic model components							
		Program goals	Program objectives	Reach	Eligibility	Program resources	Program activities	Program outcomes	Program context
Program documents (n = 15)	Initial Program Description (2013)	Not consistent	Absent	Not consistent	Absent	Not consistent	Absent	Not consistent	Absent
	Program Curricula (2015‐2018)	Absent	Absent	Absent	Absent	Not consistent	Not consistent	Absent	Absent
	Program Goals and Objectives (2016)	Not consistent	Not consistent	Absent	Absent	Not complete	Not complete	Absent	Absent
	Program Implementation Evaluation (2016)	Not consistent	Not consistent	Not complete	Absent	Not complete	Not consistent	Not consistent	Absent
	Program Referral Guide (2017)	Absent	Absent	Absent	Complete for youth only	Not complete	Absent	Absent	Absent
	Program Information for Patients and Families (2016)	Not consistent	Not consistent	Not complete	Absent	Not consistent	Not complete	Absent	Absent
	General Information for Youth and Families (2016)	Not consistent	Absent	Not complete	Complete for youth & families	Not consistent	Not complete	Absent	Absent
	Overall judgment after document analysis	Not consistent	Not consistent	Complete for youth & families		Not consistent or complete	Not consistent or complete	Not consistent	Absent
Stakeholder surveys (n = 13)	Survey questions	What are the goals & objectives of the IIPT?		Who should the program target?	No further information required	Who and what help accomplish the objective(s) of the program?		What are the effects of the program?	Context Analysis
	Overall judgment after survey analysis	Still not consistent		Complete for youth & families		Not consistent		Priority setting	Not consistent
Stakeholder focus groups (n = 6)	Focus group guiding questions	Is each component representative of the current program?							
	Overall judgment after focus groups	Complete		Expanded to include school personnel		Causal mechanisms clarified		Validated	Complete

#### 2.4.2 Step 2. Conceptual framework development

The purpose of developing the conceptual framework, the second step of the 3‐step logic analysis methodology, is to examine the intervention's main components and determine whether the optimal conditions have been assembled to achieve the desired outcomes. The aim is not to complete a systematic synthesis of the literature, but instead to create a representative synthesis of the most recent and meaningful evidence across various fields upon which the scientific validity of the logic model is examined.^26^, ^29^ To develop the conceptual framework, the 6‐stage scoping review process described by Levac et al^36^ was followed and included the stages outlined in Table 1. A scoping review was the evidence synthesis method chosen as it summarizes a range of evidence in order to convey the breadth and depth of a field.^36^ As suggested in logic analysis methodology, review studies were favored.^26^ Further details about each scoping review stage are provided below.

##### Identifying the research question

The research question identified by the expert panel was founded in the primary objective of the IIPT, as identified in Step 1 of the logic model methodology. More specifically, the following question guiding the search: “What components should an IIPT designed for youth with pain‐related disability adopt to promote self‐management, self‐efficacy and participation in age‐appropriate meaningful activities?”

##### Identifying relevant studies

MEDLINE, CINAHL, and PsycInfo electronic databases were consulted using the following key words: chronic pain; pain‐related disability; chronic conditions; disability; pediatric* or pediatric*, self‐manag*; self‐efficacy; participation. The target population was broadened to include youth with chronic conditions and disabilities for which pain is an important symptom, along with those with pain‐related disability. It has been argued that youth with chronic conditions and disability share more comparable challenges than differences and that disease‐specific orientations minimize the efficiency with which solutions for these challenges can be identified.^37^

##### Study selection

To be included, studies had to incorporate youth, aged 12‐18 years (as per the age inclusion criteria of the evaluated IIPT), be related to self‐management, self‐efficacy, and/or participation in meaningful activity (ie, leisure, recreation, or activities that promote productivity (eg, school, work)), and have a multi‐ or interdisciplinary focus. Retrieved titles and abstracts were screened by two reviewers for relevance. Entire manuscripts were then examined. Reference lists were inspected, yet no additional studies were identified. Once completed, original manuscripts cited in the review studies were scanned for additional relevant information.

##### Charting the data

A data extraction form (as per the categories outlined in Table 3) and procedures were developed and validated by the research team. Once consensus was achieved, the extraction process was completed by KH.

**TABLE 3:** Summary of studies retained for conceptual framework development

Authors & publication year	Country	Study design	Study aim	Population characteristics	Feature of included studies	Key findings
Self‐management interventions (SMI)						
Stinson et al (2008)^41^	Canada	Systematic Review	To critically appraise the evidence on effectiveness of Internet‐based SMI on health outcomes in youth with chronic conditions.	Children and adolescents (6‐18 y). Asthma, recurrent pain, encopresis, traumatic brain injury, obesity.	7 randomized control trials, 1 pilot randomized control trail, and 1 quasi‐experimental study.	Internet‐based SMI have demonstrated some evidence improving symptoms and disease self‐management yet are inconclusive in whether as effective as in‐person individualized or group interventions.
Lindsay et al (2011)^40^	Canada	Integrative Review	To synthesize findings from empirical studies examining influential factors of adolescents’ self‐management of chronic illness.	Adolescents and young adults (12‐20 y). Diabetes, asthma, spina bifida, inflammatory bowel disease, juvenile idiopathic arthritis.	34 studies, 16 qualitative, 14 quantitative, and 4 mixed‐methods designs.	Psychosocial factors (eg, self‐efficacy), parent involvement, and knowledge about illness are important facilitators. Youth self‐management skills should be assessed, along with their social and developmental context to identify supports.
Lindsay et al (2014)^39^	Canada	Systematic Review	To systematically assess the effectiveness of SMI for school‐aged children with physical disabilities.	Adolescents and young adults (13‐24 y) Children and adolescents (2‐18 y) Spina bifida, juvenile rheumatoid arthritis, juvenile idiopathic arthritis	2 randomized control trials; 4 before and after designs.	Intervention components should include knowledge about condition, medication management, psychosocial factors (eg, self‐efficacy). Parental involvement can be a barrier to self‐management and should be carefully assessed.
Sattoe et al (2015)^38^	Netherlands	Systematic Review	To provide a systematic overview of the SMI for young people with chronic conditions.	Children (7‐11 y) and adolescents (12‐18 y) Asthma, diabetes, cancer, chronic fatigue, chronic pain, chronic respiratory conditions, inflammatory bowel disease, juvenile fibromyalgia, juvenile idiopathic arthritis, migraine, physical disabilities, sickle cell.	45 randomized control trials, 29 cohort studies, 3 cross‐sectional studies, 3 qualitative, 5 mixed‐methods, 1 case study, 26 pilot evaluations.	Role and emotional management should be included in SMI, along with medical management. Parents can either facilitate or hinder youth self‐management. Experiential learning, peer learning for others, and mastery experience strategies are appropriate pediatric SMI. Developmental factors need to be considered.
Bal et al (2016)^55^	Netherlands	Systematic Review	To systematically explore the effectiveness and effective components of SMI.	Children to young adults (7‐25 y) Asthma, diabetes, cystic fibrosis, cancer, HIV, sickle cell, spina bifida, hemophilia, juvenile fibromyalgia.	42 randomized control trials.	SMI should focus on medical, emotional, and role management in the context of youth's daily lives. Peer support stimulates self‐efficacy. Online peer support could improve self‐efficacy, problem‐solving, and coping behaviors.
Lindsay, Kolne, Cagliostro (2018)^45^	Canada	Systematic Review	Synthesis and review literature on the impact of electronic mentoring for children with disabilities	Children to young adults (12‐26 y). Rheumatic disease, juvenile arthritis, cerebral palsy, spina bifida, muscular dystrophy, pediatric transplant, visual impairments, chronic pain.	3 RCTs, 7 surveys, 1 case study, 1 feasibility study.	Electronic mentoring is effective for children and youth with disabilities in improving career decision‐making, self‐determination, self‐management, self‐confidence, self‐advocacy, social skills, attitude toward disability, and coping with daily life.
Self‐efficacy						
Cramm et al (2013)^56^	Netherlands	Cross‐sectional study	To investigate the influence on general self‐efficacy perceived by adolescents with chronic conditions and parents on quality of life.	Adolescents, and young adults (12‐25 y) and their parents Diabetes, juvenile rheumatoid arthritis, cystic fibrosis, urology conditions and neuromuscular disorders.	Not applicable.	Interventions aimed at improving general self‐efficacy should include activities that seek to enhance confidence and the ability to deal effectively with difficult and unexpected events.
Johnson et al (2015)^44^	United States (US)	Cross‐sectional study	To determine the preferred methods for health information among youths with chronic conditions and their relationship to healthcare transition readiness, self‐efficacy, and medication adherence.	Children and adolescents (6‐16 y) Diabetes, musculoskeletal conditions, cerebral palsy, heart disease, neurological and gastrointestinal condition.	Not applicable.	Youth with chronic conditions receive their health information from physicians/nurses, parents/family, and the Internet. A range of health information should be considered to include those that deliver it directly to the patient, the family/parent, including the Internet, allowing youth to select their preferred method.
Molter & Abrahamson (2015)^57^	United States	Literature Review	To investigate the relationship among self‐efficacy, transition, and health outcomes.	Children, adolescents, and adults (6‐55 y). Sickle cell.	20 studies of various unspecified designs.	Knowledge of condition, body awareness, and spirituality are factors that affect self‐efficacy. Journaling, self‐awareness, scripture reading, and prayer activities can increase feelings of self‐efficacy. Experiences of acting independently and developing patient‐health provider partnerships are important. Education, counseling, and advocacy interventions to the broader public could be used to decrease stigmatization.
Kalapurakkel et al (2014)^42^	United States	Cross‐sectional study	To examine pain self‐efficacy and pain acceptance in relation to functioning in pediatric headache patients.	Children and adolescents (8‐17 y); Headache.	Not applicable.	Higher levels of self‐efficacy are associated with improved school functioning, fewer depressive symptoms, and lower disability levels, higher self‐esteem and fewer somatic symptoms.
Tomlinson et al (2017)^58^	Canada	Literature Commentary	To examine the resilience mechanism of pain self‐efficacy.	Children and adolescents.	Not specified.	Exposure to and mastery of feared activities reinforces self‐efficacy. Generalizing prior successes that highlight mastery and increase confidence can enhance pain self‐efficacy. Mindfulness and biofeedback are also helpful modalities. The identification of valued goals and utilizing graded exposure techniques to previously avoided activities promote self‐efficacy.
Participation						
Pinquart & Teubert (2011)^59^	Germany	Meta‐analysis	To compare the levels of academic, physical, and social functioning of children and adolescents with chronic physical diseases with those of healthy peers.	Children and adolescents (under the age of 18 y) Arthritis, asthma, cancer, chronic fatigue, cystic fibrosis, cerebral palsy, inflammatory bowel disease, headaches, diabetes, hemophilia, epilepsy, sickle cell, spina bifida.	954 studies designed not specified.	Sports and leisure activity counseling should be available to guide these youth. Teachers and coaches should promote participation in sports to improve physical functioning. School functioning can be improved with school accommodations. Group social skills training provides youth with strategies to deal with teasing and bullying.
Anaby et al (2015)^46^	Canada	Scoping Review	To identify and analyze research evidence regarding the effect of the environment on community participation of children with disabilities.	Children, adolescents and young adults (5‐21 y). Cerebral palsy, physical disabilities (with restricted mobility due to neurological or musculoskeletal disorders), acquired brain injury, autism spectrum disorder, Down syndrome.	31 studies; 17 qualitative, 10 qualitative, review 3, 1 mixed‐method design.	Negative attitudes within the communities can be a barrier to participation. Parental involvement and advocacy can influence on social functioning, participation, and friendship development. Peers, teacher, and service provider support fostering participation. Parental over‐protectiveness and stress can limit participation. Parental education about recreation activities and advocacy supports participation.
Adair et al (2015)^48^	Australia	Systematic review	To critically appraise studies aimed at improving participation outcomes of children with disabilities.	Children and adolescents with disabilities (5‐18 y) such as cerebral palsy, developmental coordination disorder, autism spectrum disorder, arthrogryposis, intellectual disabilities.	7 randomized control or nonrandomized trials	Tailored programs using both individual‐ and group‐based approaches can enhance participation. Coaching approaches focused on mutually agreed upon goals are effective. Practice of desired behaviors in a social context is proven useful.
Forgeron et al (2018)^60^	Canada	Systematic Review	To identify the psychosocial interventions found to be most promising in their effectiveness in improving social functioning outcomes of children and adolescents with a wide range of chronic physical health conditions.	Children and adolescents (5‐18 y) with diabetes, epilepsy/seizures, cerebral palsy, spina bifida, inflammatory bowel disease, burn scaring, chronic respiratory condition.	13 studies; 10 nonrandomized control trials, 3 randomized control trials.	Most improvements in social functioning stemmed from interventions that focused on a broad range of social skill development rather than solely on communication about condition with peers. Interventions that consisted of more than one session targeting social functioning were more promising. A paucity of evidence exists on effective interventions.
Jones et al (2018)^49^	Canada	Narrative review	To review selected studies that have made an impact on the field of school functioning in children and adolescents with chronic pain.	Children and adolescents (8‐18 y) with chronic pain such as abdominal, myofascial, neuropathic, limb, back pain, headache.	13 nonrandomized control trials.	Evidence suggests that psychological factors (depression and anxiety), social factors (peer relationships, perception of teachers support, parent protectiveness), physiological factors (sleep disturbance), and cognitive factors (self‐efficacy, memory, and attention deficits) may interact to influence school functioning.
Ideal context						
Stahlschmidt et al (2016)^12^	Germany	Review	To present an international perspective on the structure and components of pain rehabilitation programs worldwide.	9 different programs from 4 different countries.	15 descriptive or nonrandomized studies.	Specialized rehabilitation programs for disabling chronic pain conditions worldwide have similar admission criteria, structure, and therapeutic orientation. Differences in exclusion criteria impede program comparability.
Miró et al (2017)^61^	Spain	Cross‐sectional study design using surveys	To identify the features’ current chronic pain programs and describe the feature required to achieve an ideal state.	136 pediatric pain experts located in 12 different countries.	Not applicable.	Staff should be multidisciplinary, with research and formal specialty training available. A wide variety of treatment options should be offered and publicly funded.
Harrison et al (2019)^7^	United States, Belgium, Stockholm	Review	To present an overview of rehabilitation interventions for children and adolescents with chronic pain and to inform clinicians on the innovative treatment delivery and patient outcomes.	Not applicable.	Systematic review, meta‐analyses, clinical trials with sample >20, clearly describing the intervention.	Patients who have been unsuccessful at outpatient treatment are targeted. Must include three or more disciplines housed within the same facility (eg, pain specialist, psychologist, and physical therapist) who work in an integrated manner to provide treatment. Patient must participate in exercise‐based therapy and psychological interventions. The aim is to improve function across domains. Variability exists in program structure, organization, frequency of treatment across disciplines, treatment model (inpatients vs. day‐hospital), and length of stay.

##### Collating, summarizing, and reporting the results

Data were coded, categorized, themed, and then culminated into a table format (see Table 4). An initial draft of the conceptual model was presented and discussed with the expert panel to explore the meaning, clarity, and consistency of the thematic interpretation.

**TABLE 4:** Conceptual framework

Logic model components	Self‐management interventions	Building self‐efficacy	Fostering participation
Program objectives			
Program goals and objectives	Role, and emotional and medical self‐management relative to developmental expectations should be integrated within youth's daily life and relevant social contexts^38^, ^39^, ^55^		
Program reach and eligibility	Parent involvement should be carefully assessed^38^, ^39^, ^40^ Education should extend beyond youth with chronic conditions and parents, to include peers and teachers^39^, ^47^		Education initiatives should target peers, classmates, teachers, and community leaders (eg, coaches)^46^, ^49^, ^59^
Program activities	Psychoeducation, combining information and skills training, is the focus of self‐management interventions^38^, ^55^ Parent education, parent‐to‐parent support, and using parent coaching approaches are effective in fostering independence in youth self‐management^39^ Experiential approaches, varying delivery methods (group, individualized, Internet‐based), peers learning opportunities, and skill mastery experiences should be provided^38^, ^39^, ^40^, ^41^ Communication, assertiveness, and advocacy training are a need identified by youth to promote shared decision‐making with professionals^39^, ^41^ Opportunities for youth to create their own patient‐professional relationships can be enriching^41^ Peer‐to‐peer learning and mentoring is an emerging model showing promise^45^	Activities that build independence, life, and leadership skills should be promoted^56^ Opportunities for youth to create their own patient‐professional relationships can be enriching^44^, ^56^ Self‐awareness (eg, journaling), self‐directed learning (eg, web‐based resources), and spiritual program activities, using a variety of learning methods and mediums (eg, health professionals, parents, Internet‐based modules) should be included^44^, ^45^, ^57^ Biofeedback, self‐regulation, relaxation, mindfulness, cognitive‐behavioral therapy, value‐based goal identification nurture self‐efficacy^58^ Successful accomplishment of assigned tasks and generalization of prior successes, and graded exposure to fear‐eliciting activities are also beneficial^58^	Individualized and group‐based interventions are effective when combined^48^ Physical and leisure activity selection should be guided by mutually agreed upon participation goals and identified through coaching approaches^48^ Training parents and youth on how to advocate for social inclusion and how to adapt and modify the activity and environment are effective strategies to minimize participation barriers^46^ Sport and leisure activity counseling and social skills training should be available^48^ Coaching on how to communicate about the condition and the supports required may be beneficial for this population in peer and school settings^46^, ^48^, ^49^ More complex age‐specific in‐person sessions expanding social skills training to peer interactions, conflicts (eg, bullying), and intimate friendships may also be beneficial for older adolescents^59^, ^60^
Program outcomes	Increased knowledge and skills in problem‐solving, decision‐making, and advocacy have been described^38^ Improvements in self‐efficacy, psychosocial well‐being, and family functioning, along with reduction in social isolation, school absenteeism and pain have been demonstrated^41^ Reduced family and parent burden, reducing healthcare utilization, and improving overall health outcomes and quality of life have also been reported^38^	Benefits to physical, emotional, and school functioning have been recognized^42^ Self‐efficacy has been identified as a key contributor to chronic disease self‐management, to promoting of long‐term behavior change, to improving the appropriateness of healthcare utilization practices, and to enhancing health quality of life^43^	Participation improved academic performance, social interactions, mental and physical health, and helps develop life purpose and meaning^46^, ^62^
Creating the ideal context			
Program resources	Program should be publicly funded^61^ A variety of health disciplines with specific training and expertise in pediatric pain^7^, ^12^, ^61^ A clinical and research training role, along with a public education (eg, school personnel) and advocacy mandate should be fulfilled by the program^61^ Youth with variety of pain conditions, regardless of the type and origin, and their parents should be targeted^7^, ^12^, ^61^		

##### Consultation

As identified in Table 1, the expert panel members were involved in the scoping review in the initial three stages of the review, provided consultation throughout the process, and assisted in the re‐interpretation of the data in the context of IIPT.

#### 2.4.3 Step 3. Evaluation of the program theory

The third and final step of the logic analysis methodology consisted of comparing the constructed logic model with the developed conceptual framework.^26^ Moreover, this comparison examined the scientific validity of the program theory,^29^ identified program gaps, and highlighted potential program improvements.^26^ This step was completed collaboratively with the expert panel. It began with rereading of the program logic model, the appraisal of its components, and the examination of their relationship with those identified in the conceptual framework. Discrepancies and connections were initially identified by two members of the research team. Prior to the expert panel meeting, a compiled list of identified program strengths and weaknesses, copies of the logic model, and the conceptual framework were distributed electronically to members. At the meeting, the discrepancies were debated in relations to the members’ experiential knowledge. Recommendations upon which consensus was achieved were then shared with hospital leadership.

## 3 FINDINGS

### 3.1 Logic model construction

#### 3.1.1 Program documents

Fifteen key program documents and 13 stakeholder surveys were used to construct the draft logic model. Although the documents contained many important program details, when closely compared, inconsistencies emerged (see Table 2). Different program objectives were noted across documents. For example, stated goals/objectives focused on youth returning to age‐appropriate activities, or on the resumption of participation in social roles in various contexts (eg, students at school); some specified goal achievement, despite pain, while others promised a decrease in pain over time. Program resources, related to clinical disciplines, also varied. Program activities were described as a function of these disciplines, which, in some cases, varied depending on the cohort and the chosen service model (eg, individual‐focused versus group‐based). Although program outcomes were present in select documents, they were not linked to the program activities or resources, and their relationships with the program objectives were unclear. The anticipated causal mechanisms between the activities and the expected program outcomes were unidentifiable. Finally, contextual factors were scant.

#### 3.1.2 Expert panel surveys

Survey responses assisted in further elaborating the logic model components, although discrepancies remained. A synthesis of the program resources, activities, causal mechanisms, and expected outcomes as perceived by the expert panel revealed that, similar to the document analysis, most expert panel members (ie, clinicians) described program activities as a function of the disciplines (see Appendix S2). Furthermore, perceived mechanisms varied and were considered unique to each activity. The service model (ie, group‐vs. individual‐based), the program intensity, and preprogram activities were viewed to be important contributors by some. Despite these added details, the relationship between the mechanisms and outcomes remained ambiguous (see Table 2). Contextual factors were also identified in the survey responses (see Appendix S3). Internal factors were linked to program structure and team dynamics, while external factors were related to building community‐based partnerships and securing future program funding. Although these factors helped to further understand the context and the conditions deemed essential for success, questions remained.

#### 3.1.3 Group meetings

At the first expert panel group meeting, a new program objective drafted and distributed prior to the meeting was validated. The program objectives became “To provide youth with pain‐related disability and their parents the knowledge, skills, and tools to self‐manage their pain, build their self‐efficacy, and promote their participation in meaningful activities, despite their pain.” Furthermore, based on expert panel discourse as per the member below, the program reach was extended to include school and community personnel.

Our target population should include parents and the school, but also others in their community environment. (Clinician 1)

Some activities and processes were omitted, while others were added, or further detailed. Program activities, which provided support, most valued by parents and youth were underscored.

I think two things are absolutely fundamental in this program: the education group sessions and the connections you have with the other participants. (Youth 2)

Youth also recognized activities that should be added to further improve their outcomes. Such activities focused on self‐advocacy and the need to facilitate their transition back to their community following the program. The expected outcomes were adjusted and further elucidated based on panel member's experience.

In terms of long‐term outcomes, it should be how much knowledge is retained. Because if you can refine the application of that knowledge; and once you build routines, you’ve found a way to make it work for you. (Youth 1)

**FIGURE 2:**
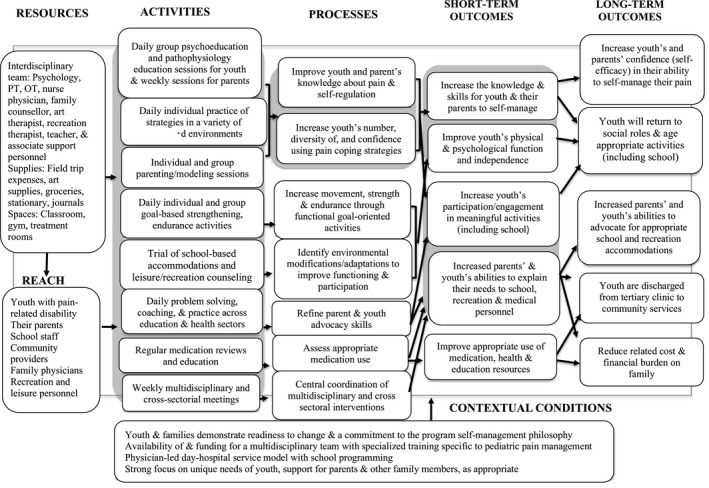
Expert panel agreed upon logic model

Finally, contextual factors believed to be essential for program success were discussed, and agreement was reached. These factors were associated with the preprogram screening, access to specialized health human resources, and participant characteristics. Figure 2 illustrates the final agreed upon logic model.

### 3.2 Development of the conceptual framework

#### 3.2.1 Scoping review results

Table 3 outlines the details of the 19 articles selected for the conceptual framework development and the deductive framework used to extract the data. All population samples included children and adolescents with a variety of disabling conditions for which pain is an important symptom.

#### 3.2.2 Conceptual framework summary

Table 4 synthesizes the salient evidence of the conceptual framework, its relationship with both the logic model components, and the themes supportive of the program's key objectives. Further description is provided below.

##### Promoting self‐management

Self‐management, defined as a person's ability to acquire and apply the skills and knowledge to manage their symptoms, is learned with the support of one's family, community members (eg, friends, peers, teachers, coaches), and healthcare professionals.^38^ Chronic conditions are experienced within the perspective of everyday life contexts (ie, peers, family, school, occupation, leisure, community).^38^, ^39^ Although medical management is important, emotional coping and role (social participation, occupation) management should also be considered.^40^ Effective medical self‐management is contingent on youth acquiring independence, knowledge, and skills.^41^ Psychoeducation and skills training are the cornerstones of self‐management programs.^7^, ^41^ Parental education and parent‐to‐parent support are effective in addressing the gradual shift of self‐management responsibilities to youth.^39^ Support from social networks, including peers, has also emerged as a facilitator.^38^, ^39^, ^40^, ^41^ Many additional effective activities and promising emerging approaches are presented in the conceptual framework (see Table 4).

##### Building self‐efficacy

Self‐efficacy, defined as a youth's confidence in their ability to function effectively while in pain,^42^ is critical to self‐management, to appropriate healthcare utilization practices, and to enhancing health‐related quality of life.^43^ Effective activities for building self‐efficacy were highlighted in the framework (see Table 4). Appealing to youth's preferred information seeking practices is considered pivotal to the process, with web‐ and application‐based resources holding promise for this population.^44^, ^45^

##### Enhancing participation in meaningful activities

Participation, defined as one's involvement in life situations (eg, education, employment, recreation, and community living), is an important pediatric rehabilitation outcome.^46^, ^47^ Social supports (eg, school personnel, peers) are important facilitators to achieving participation.^46^ Moreover, effectively communicating about one's condition and requesting the supports required within various contexts (eg, in school, with peers) are important skills for increasing participation.^46^, ^48^, ^49^ Other associated activities are presented in Table 4.

##### Creating the ideal context

Contextual conditions essential for program success were also found in the literature. Admission criteria across IIPT programs worldwide are similar, of which, pain impacting function, and youth and parent commitment to a self‐management approach dominate.^7^, ^12^ Other contextual factors are highlighted in the conceptual framework (see Table 4).

### 3.3 Evaluating the intervention theory

When detailed IIPT components, their links, and anticipated outcomes were systematically compared to the conceptual framework, generally speaking, the scientific evidence supported the program theory plausibility. Furthermore, interconnectivity between the three IIPT program objectives was illustrated. Below the IIPT program, strengths are presented, followed by recommendations for improvements.

#### 3.3.1 IIPT strengths

Regarding refining the self‐management intervention for youth, our IIPT intervention aligned well with the evidence contained in the conceptual framework. As per the evidence, psychoeducation was acknowledged as a valued tenet of the program. Many teaching approaches (eg, peer learning) recognized as effective were incorporated in the program group activities and included opportunities for practice in real‐life environments (eg, classroom, community field trips). These peer‐learning moments were highly valued by expert panel parent and youth members and recognized as pivotal in achieving positive outcomes. However, a need to incorporate additional community‐focused transition opportunities was underscored by both parents and youth, and by the scientific evidence reviewed.

In relation to building self‐efficacy, our IIPT program also performed well against the scientific evidence of the conceptual framework. In addition to family counseling and individual psychological interventions, many targeted activities identified as beneficial (eg, self‐awareness, self‐reflection) in the evidence were already incorporated in the IIPT. Moreover, the inclusion of community‐based activities (eg, field trip, leisure planning) in the IIPT, designed to foster problem‐solving, decision‐making, and self‐management skills and their generalization to real life, was strongly supported by the scientific evidence and the experiential knowledge of the youth expert panel members. However, youth panel members also requested even further guidance on the safe return to such activities postdischarge.

With respect to fostering participation in meaningful activity, the IIPT included several components deemed effective based on the evidence. Sports, recreation and leisure counseling, advocacy education, and youth and parental training in activity and environment modifications were activities already incorporated in the IIPT and for which conceptual framework scientific support existed. Transition meetings with school personnel, part of the current program discharge process, were acknowledged by youth and parent expert panel members as an opportunity to foster collaboration with teachers, which coincided with the conceptual framework evidence. Youth expert panel members not only valued these meetings, they requested additional tools to further facilitate their ongoing advocacy initiatives in this context postdischarge.

Finally, concerning creating an ideal context to achieve the anticipated program outcomes the IIPT fulfilled many of the prerequisite conditions identified in the conceptual framework. When compared, the IIPT admission criteria, key program features, and team memberships shared many similarities with studies included in the conceptual framework.

#### 3.3.2 IIPT improvements

When comparing the logic model to the conceptual framework, three main areas of improvement associated with the reach, activities, and processes of the evaluated IIPT were presented to the expert panel for consideration. First, the importance of adopting a developmental lens to the acquisition of knowledge and skills aligned with the expectations of different age groups was recognized. Although the IIPT integrates school‐based, sports, leisure, and recreation activities, the evidence supported incorporating sessions addressing topics such as vocation and work, independent living (eg, housing), and the management of intimate relationships, for older youth (ie, 16‐18 years). Youth expert panel members also advocated for postprogram support associated with the quickly changing responsibilities and mounting societal expectations inherent to this age group. To incorporate this empirical and experiential knowledge, the inclusion of developmental goals to the already existing goal‐setting process was suggested. The conceptual framework also highlighted the need to expand the reach of the program to include youth's broader social networks. Enhancing peer support through educating classmates and school personnel on pain‐related disability and on how to support to those suffering from this condition was recommended. Expert panel clinicians, youth, and parents’ members alike acknowledged this missing pillar in the IIPT. Finally, the conceptual framework highlighted emerging evidence supporting the use of the web and application technology. Although the technological trials have been limited to one or two of the IIPT components (eg, cognitive‐behavioral therapy), these technologies hold promise for families for whom access to trained professionals, distance from care facilities, and long waiting times are major barriers. However, web‐based expansion of any of our program component was not acknowledged or recognized as a gap by our expert panel. Upon review of these IIPT improvement recommendations and in light of the organizational constraints raised by the health manager expert panel member, the panel provided the following recommendations to the hospital leadership team: (a) expand information provided to older adolescents to incorporate vocation, work, independent living, and relationships; (b) incorporate self‐management goals tailored to the developmental spectrum; and (c) broaden the psychoeducation to involve peers and school personnel.

## 4 DISCUSSION

The purpose of this article was to detail the logic analysis methodology and to share the findings of the program theory testing of an IIPT using this approach. As a collaborative IKT approach, this evaluation methodology proved helpful in many ways. First, logic analysis provided an opportunity to create a shared understanding of the complexity of IIPT among stakeholders, highlighting previously unidentified intervention and context interactive mechanisms. Stakeholder engagement was critical in ensuring the accuracy, validity, and the integrity of the implemented IIPT description. Furthermore, stakeholders’ reflections, in particular those of youth and their parents, were crucial in establishing those causal mechanisms and activities most valued. Through this value‐based process, mechanisms were identified where interactions between the invention and the context occurred. Complex interventions, like IIPT, are built on a number of components, which may be dependent and interdependent, and where interactions between the intervention and the context exist.^50^ It has been previously suggested that the effectiveness of these interventions may rest in the interaction between the intervention components (eg, psychoeducation) and the context (eg, group milieu, staff interactions, real‐life situation). To date, the exploration of these interactive intervention‐context mechanisms have been rare.^12^ The logic analysis methodology presented a standardized approach which not only helped theorize this complex intervention, but also assisted in acknowledging intervention‐context interactive mechanisms (eg, psychoeducation in peer‐supported environments), as a result of the engagement of the target population.

Secondly, the logic analysis process assisted in unveiling health professionals’ beliefs about the causal mechanisms thought to contribute to the achievement of the anticipated outcomes. It provided an opportunity to weigh these assumptions against two important sources of validity: scientific evidence and youth and caregivers’ experiential knowledge and values. More importantly, both these sources failed to confirm clinicians’ assumptions of discipline and activity‐specific mechanisms. In evaluation research, it has been recognized that the mechanisms of change are not so much linked to the interventions per se, but instead to the participants’ reasoning and responses generated by the activity and the context which lead to the outcomes of interest.^51^ Further exploration of youth and their parents’ reasoning and responses to IIPT activities and the program as a whole, and within different daily contexts (eg, school, home), may represent valuable new avenues of research in this field.

Thirdly, the conceptual framework used a recognized evidence review method and presented a synthesis of current evidence to the expert panel members. This evidence‐informed framework stimulated practice reflection and comparison with experiential knowledge and values. As such, logic analysis presented an innovative way to integrate IKT, addressing the persisting knowledge‐to‐practice gap in pediatric rehabilitation. Discovering scientific evidence to support many of the causal mechanisms of the evaluated program and gaining awareness of those components most valued by youth and their families were noted by clinician expert panel members to be most enlightening part of this collaborative process. Whether this reflective process and increased awareness of the evidence prompt behavior and practice change in clinicians will require further investigation.

Engaging stakeholders in logic analysis has been previously recommended.^29^ Particularly unique in our application of this methodology was the involvement of patients (ie, youth with pain‐related disability) and their caregivers. The premise of engaging patients beyond the level of research subjects reflects a growing desire for more ethical, democratic, and moral practices.^52^ However, the absence of parent and youth voices in the published evaluation of pediatric pain rehabilitation interventions, including IIPT, is a gap recognized by many.^17^, ^19^, ^38^, ^53^ In our evaluation, their engagement resulted in identifying youth and their parents’ program expectations, as well as recognizing their ongoing challenges following program discharge. Also noteowrthy was the causal mechanisms identified by youth and parent expert panel members, as experiential knowledge was acknowledge in the scientific evidence incorporated into the conceptual framwork. Building this shared understanding within the expert panel proved valuable in later prioritizing program refinements. Furthermore, organization constraints highlighted by the health manager provided important insight into selecting recommendations that were feasible to implement within the program context.

Specific evidence‐informed practices and strategies to foster stakeholder engagement were incorporated into this logic analysis methodology. Targeted activities included (a) choosing a sample of parents and youth who have used the services,^19^ (b) creating clearly defined roles, responsibilities, and expectations for the expert panel members and research team,^54^ (c) engaging stakeholders early and throughout in the evaluation process,^16^, ^54^ (d) providing training on evaluation principles,^53^, ^54^ (e) ensuring regular interactions with the panel to foster mutual understanding among members,^15^ (f) embracing a variety of communication technologies to promote participation and discussion,^19^ and (g) distributing discussion materials prior to the meeting.^15^

Despite our best efforts, this study should be interpreted with some limitations in mind. First, the nonequivalent numbers in each of our stakeholder groups on our expert panel may have biased our results and may have created a power imbalance in favor of clinicians in the group discussions. A variety of data collection methods were, however, used, incorporating anonymous strategies (eg, electronic surveys) to ensure authentic perspective were expressed by expert panel member, decreasing social desirability biases. Second, despite expansive recruitment efforts, limited diversity was evident in our expert panel membership. Although youth and parents were representative of the population using this program, other recruitment strategies should be explored if this methodology is expanded to interventions servicing a more cultural and ethnic diverse population. Third, the inclusion of expert panel members into the conceptual framework development could be enhanced. In previously described logic analysis processes, the conceptual framework phase was completed by the evaluator only. Although the expert panel members were included in many stages of the conceptual framework construction, incorporating stakeholders in the data extraction and theming processes of the scoping review could be added if appropriate oversight was provided.

Theory‐based evaluation provided an opportunity to further detail the causal path of IIPT rehabilitation intervention, leading to a better understanding of these interventions, and evaluated the plausibility of the program theory in achieving its anticipated outcomes. Stakeholders were implicit to this process. The methods presented in this article, where scientific and experiential knowledge were weighed in a similar manner, provided a collaborative, pragmatic, and realistic approach, representative of the clinical environment in which most healthcare providers conduct evaluation. Engaging stakeholders, including parents and youth, in the logic analysis represents a catalyst for better understanding complex of pediatric pain rehabilitation interventions, such as IIPT, and their evaluations. Furthermore, it represents a novel IKT method to narrow the ongoing knowledge‐to‐practice gap existent in the field.

## CONFLICT OF INTEREST

The authors have nothing to declare.

## AUTHORS CONTRIBUTIONS

This study was conducted by Karen Hurtubise as a requirement for the Doctor of Philosophy Degree. Karen Hurtubise's contribution to this manuscript included its conceptualization, the literature and scoping review, the data analysis, creation of the conceptual framework, the presentation of the findings, and the writing of this manuscript. Dr Astrid Brousselle provided methodological evaluation expertise in the development of this study, supervision, and guidance during the various stages of the study. She also viewed all results and provided feedback on the manuscript prior to submission. Dr Chantal Camden provided expertise in stakeholder engagement processes and integrated knowledge translation strategies throughout the study, validated the results at the various stages of the study, reviewed, and provided feedback on the manuscript prior to submission. She also provided supervision and guidance throughout the study.

## Supporting information

Supinfo 1

Supinfo 2

Supinfo 3
