# Supplementary material for: Using collaborative logic analysis evaluation to test the program theory of an intensive interdisciplinary pain treatment for youth with pain‐related disability
Source: Paediatr Neonatal Pain. 2020 Apr 23;2(4):113–30. doi: 10.1002/pne2.12018 (PMC8975192; doi:10.1002/pne2.12018)
Supplement: Supplementary file 1 — Supinfo 1 [file PNE2-2-113-s003.docx]

**Supplementary Material 1. Expert Panel Survey**

**Program Model Survey**

| **Purpose:** This questionnaire includes 8 short answer questions that will assist in providing us with some information about your thoughts on the current intensive rehabilitation program to help us better understanthe program what it is designed to achieve, how it achieves it and what are the things that have or could influence it. The information will be used to create a visual representation of the program (also known as a logic model) that will be discussed at our next meeting.  **Instructions:** Carefully read the question and fill in the space below. Please note there are no wrong answers and all information is welcomed.  **Q1. According to you, what is the objective(s) of the intensive pain rehabilitation program? Please give examples on how that objective could be observed.**  **Q2. According to you, what are the activities of the intensive rehabilitation program that help accomplish this objective? Please be as specific as possible in naming the activity components.**  **Q3. How do you think these activities contribute in accomplishing the objective(s) of the program? Please describe the links you think exist between what the activities do and why you would expect a specific outcome to result.**  **Q.4 According to you, what are the strengths of the intensive pain rehabilitation program?** *Strengths are aspects of the program you are pleased with and that currently contribute to the quality of the services offered.*  **Q.5 According to you, what are the weaknesses of the intensive pain rehabilitation program?** *Weaknesses are aspects of the program that need to be improved to increase the quality of the services provides.*  **Q6. According to you, what opportunities exist that could positively improve the quality of services of the intensive pain rehabilitation program?** *Opportunities are elements within the hospital or in the community at large that favor the development of the intensive pain rehabilitation program and can help to improve the quality of the services provided.* |
| --- |

**Q.7 According to you, what are the treats that could negatively impact the quality of service provided by the intensive pain rehabilitation program?**

*Threats are elements at the hospital or in the community at large that can hinder the quality of service of the intensive pain rehabilitation.*

**Q.8 Is there anything else you wish to tell us about the program that would help us better understand it?**

Thank you for your thoughts!
