# Supplementary material for: Using collaborative logic analysis evaluation to test the program theory of an intensive interdisciplinary pain treatment for youth with pain‐related disability
Source: Paediatr Neonatal Pain. 2020 Apr 23;2(4):113–30. doi: 10.1002/pne2.12018 (PMC8975192; doi:10.1002/pne2.12018)
Supplement: Supplementary file 2 — Supinfo 2 [file PNE2-2-113-s002.docx]

**Supplementary Material 2. Expert Panel themes about causal mechanisms and program structure assumptions**

| **Resources** | **Activities** | **Mechanisms** |
| --- | --- | --- |
| Physiotherapy  Psychology  Medicine  Family Therapy  Occupational Therapy  Music Therapy  Art Therapy  Recreation  Nursing  Academics/School  Program Structures | Behavioral activation or physical re-activation  Improve physical endurance, tolerance, strength, flexibility and posture  Goal-oriented physical activity and home programming  Graded increase in physical activity  Flare plan development  Home Program activities  Active pain management and coping strategies  Education and training on thoughts, feelings, behaviors, acceptance  Individual and group coaching in self-management  Sleep Education  Flare plan development  Focus on mindfulness  Provision of rational for rehabilitation approach & medical closure  Medication management  Progress updates  Group and therapy session to coach parents on how to support their children  Parental pain education  Parental support  Identification and management of family issues  Ergonomic assessment and recommendations  Adaptation to activities of daily living  Sleep Education  Pacing  Development of music play-lists  Alternative to express emotions & chronic pain experience  Graded increase in leisure activity and sports  Exploration of alternative sport and leisure activities in community  Parent and adolescent support  Medication management  Assessment of learning needs  Group-based service delivery  Intense duration  Signing of daily attendance expectations  Contract pre-program | Increases activity and movement  Reduces fear avoidance  Improves fitness, endurance, and strength  Fosters reconditioning  Dampens pain signals in the brain  Facilitates adherence  Promotes improve coping  Increases energy reserves  Sets expectations for ongoing practice of learning and skills  Promotes the establishment of a routine  Improves youth’s understanding of their pain at a physiological level  Reduces fear, anxiety and depression  Increases self-efficacy  Teaches youth and their family to think and talk about pain differently  Promotes functioning and participation, despite pain  Enhances self-regulation  Increases youth and parents feeling in control  Improves treatment adherence  Improves buy-in to rehabilitation approach  Empowers family to support their child  Creates a supportive family and transition environment for youth following program  Keeps parents informed about what their child is learning  Supports youth in daily activities through to transition back into the community  Facilitates an alternate coping strategy  Promotes motivation, relaxation or distraction a needed  Self-expression  Promotes an alternate coping strategy  Self-expression of chronic pain  Provides realistic contexts in which to apply self- management knowledge and skills  Promotes transition to community activities post-program  Supports families  Enables youth to maintain school some level of academic expectations  Encourages a routine and normalcy  Assists in identifying academic and social school-based challenges  Eases transition back to community school setting  Decreases isolation  Teaches support of others and of self  Validation of the experience by others  Creation of a support network  Peer discussions and learning  Facilitates empathy towards others  Allows time for the immediate application of learnings and reinforcement  Concentrates learning and practice  Sets future expectations for daily school attendance |
