# Supplementary material for: Using collaborative logic analysis evaluation to test the program theory of an intensive interdisciplinary pain treatment for youth with pain‐related disability
Source: Paediatr Neonatal Pain. 2020 Apr 23;2(4):113–30. doi: 10.1002/pne2.12018 (PMC8975192; doi:10.1002/pne2.12018)
Supplement: Supplementary file 3 — Supinfo 3 [file PNE2-2-113-s001.docx]

**Supplementary Material 3. Expert Panel survey response context analysis**

|  | Strengths | Weaknesses | Opportunities | Threats |
| --- | --- | --- | --- | --- |
| Program structures & organization | Program intensity   - Re-establishes a daily routine - Focused learning and practice on self-management - Less disruptive to family life   Decreases stress & anxiety in youth as removed from some environments (e.g. school)  Encourages fun  Tailored for youth’s learning capacity  Development of support network for youth and parents  Integration of academic component | Lack of a standardized intake process and clear criteria  Difficulty recruiting patient  Time demands and stress on youth, family and staff   - Loss important learning and social opportunities   Complexity of intervention  Length of program  Lack of coordination of program components   - Need for clear transition early in the program - Stronger curriculum throughout program   Need for additional components/time   - Parent education about accommodations - More academic time | Streamlining and/or standardization of program  Pan-Canadian recruitment & marketing  Integration of other hospital services | Not offering program to all youth who could benefit  Competition with other programs |
| Team members and dynamic | Caring, positive, expert staff  Wholistic & comprehensive approach  Multidisciplinary approach | Team dynamics and conflict   - Communication - Lack of a shared philosophy on program components   Too many professionals involved  Lack of capacity building opportunities for program staff   - Cross-coverage within and between disciplines - Development of additional expertise |  | Loss of key staff resources |
| Building partnerships |  |  | Development of community partnership  Build in volunteer opportunities into program  Expand space available,  Creation of education and training for personnel | Lack of society knowledge and recognition of pediatric chronic pain |
| Program funding |  |  |  | Future funding  Perceived as expensive |
